# Radiosynthesis and preclinical evaluation of a ^68^Ga-labeled tetrahydroisoquinoline-based ligand for PET imaging of C-X-C chemokine receptor type 4 in an animal model of glioblastoma

**DOI:** 10.1186/s41181-024-00290-y

**Published:** 2024-08-20

**Authors:** Piyapan Suwattananuruk, Sukanya Yaset, Chanisa Chotipanich, Angel Moldes-Anaya, Rune Sundset, Rodrigo Berzaghi, Stine Figenschau, Sandra Claes, Dominique Schols, Pornchai Rojsitthisak, Mathias Kranz, Opa Vajragupta

**Affiliations:** 1 Department of Food and Pharmaceutical Chemistry and Center of Excellence in Natural Products for Ageing and Chronic Diseases, Faculty of Pharmaceutical Sciences, Chulalongkorn University, Bangkok, Thailand; 2 Molecular Probes for Imaging Research Network, Faculty of Pharmaceutical Sciences, Chulalongkorn University, Bangkok, Thailand; 3 National Cyclotron and PET Centre, Chulabhorn Hospital, Bangkok, Thailand; 4 PET Imaging Center, University Hospital of North Norway, Tromsø, Norway; 5 Department of Clinical Medicine, Nuclear Medicine and Radiation Biology Research Group, UiT The Arctic University of Norway, Tromsø, Norway; 6 Department of Microbiology, Immunology and Transplantation, Laboratory of Virology and Chemotherapy, KU Leuven, Louvain, Belgium

**Keywords:** CXCR4 receptor, Glioblastoma (GBM), ^68^Ga, Bifunctional chelator (BFC), Positron emission tomography, PET tracer

## Abstract

**Background:**

This study aimed to develop a novel positron emission tomography (PET) tracer, [^68^Ga]Ga-TD-01, for CXCR4 imaging. To achieve this goal, the molecular scaffold of TIQ15 was tuned by conjugation with the DOTA chelator to make it suitable for ^68^Ga radiolabeling.

**Methods:**

A bifunctional chelator was prepared by conjugating the amine group of TIQ15 with *p-*NCS-Bz-DOTA, yielding TD-01, with a high yield (68.92%). TD-01 was then radiolabeled with ^68^Ga using 0.1 M ammonium acetate at 60 °C for 10 min. A 1-h dynamic small animal PET/MRI study of the labeled compound in GL261-luc2 tumor-bearing mice was performed, and brain tumor uptake was assessed. Blocking studies involved pre-administration of TIQ15 (10 mg/kg) 10 min before the PET procedure started.

**Results:**

[^68^Ga]Ga-TD-01 exhibited a radiochemical yield (RCY) of 36.33 ± 1.50% (EOS), with a radiochemical purity > 99% and a molar activity of 55.79 ± 1.96 GBq/µmol (EOS). The radiotracer showed in vitro stability in PBS and human plasma for over 4 h. Biodistribution studies in healthy animals revealed favorable kinetics for subsequent PET pharmacokinetic modeling with low uptake in the brain and moderate uptake in lungs, intestines and spleen. Elimination could be assigned to a renal-hepatic pathway as showed by high uptake in kidneys, liver, and urinary bladder. Importantly, [^68^Ga]Ga-TD-01 uptake in glioblastoma (GBM)-bearing mice significantly decreased upon competition with TIQ15, with a baseline tumor-to-background ratios > 2.5 (20 min p.i.), indicating high specificity.

**Conclusion:**

The newly developed CXCR4 PET tracer, [^68^Ga]Ga-TD-01, exhibited a high binding inhibition for CXCR4, excellent in vitro stability, and favorable pharmacokinetics, suggesting that the compound is a promising candidate for full in vivo characterization of CXCR4 expression in GBM, with potential for further development as a tool in cancer diagnosis.

**Supplementary Information:**

The online version contains supplementary material available at 10.1186/s41181-024-00290-y.

## Introduction

Glioblastoma multiforme (GBM) stands as a formidable challenge in the realm of oncology, representing the most invasive primary brain cancer and presenting patients with a poor prognosis (Tykocki and Eltayeb 2018; Wu et al. 2021). GBM, with a relatively low incidence rate of less than 3.19 cases per 100,000 individuals, is known for its aggressiveness persists aggressive nature and poor prognosis. This aggressiveness persists regardless of advancements in both standard and novel therapeutic modalities that have shown promise in improving patient outcomes (Tebha et al. 2023). Diagnosing and treating brain cancer, particularly GBM, remains complicated due to rapid tumor growth and unclear targeted receptors for diagnosis and therapy. This uncertainty makes the development of effective drugs and PET tracers challenging (Herholz et al. 2012; Kracht et al. 2004; Nowosielski et al. 2014; Muthukumar et al. 2023). Metabolic activity in cells is commonly used to evaluate GBM in patients. However, the effectiveness of this conventional method in detecting tumors is limited by metabolic similarities between cancerous and normal cells, as seen with [^18^F]FDG and [^18^F]FET (Elboga et al. 2022; Waltenberger et al. 2022). Recently, [^68^Ga]Ga-PSMA and [^68^Ga]Ga-FAPI have been utilized to detect brain cancer (Borja et al. 2021; Zhao et al. 2015). Evidence suggests that prostate-specific membrane antigen (PSMA) is expressed in the neovasculature of various solid tumors, including GBM, making [^68^Ga]Ga-PSMA a potential candidate for imaging GBM (Holzgreve et al. 2021; Lith et al. 2023). However, PSMA expression in GBM is generally found in the tumor-associated neovasculature rather than in the tumor cells themselves, which may affect the sensitivity and specificity of [^68^Ga]Ga-PSMA PET imaging in GBM compared to prostate cancer (Holzgreve et al. 2021). The [^68^Ga]Ga-FAPI PET imaging target is fibroblast activation protein (FAP) which is commonly overexpressed in cancer-associated fibroblasts within the tumor microenvironment of various cancers, including glioblastoma (Liu et al. 2015). Nevertheless, more research is needed to fully establish its diagnostic accuracy and clinical utility in GBM (Yang et al. 2023).

In light of these challenges, there is a critical need for targeted imaging tools specifically designed for brain cancer, particularly GBM. The C-X-C chemokine receptor 4 (CXCR4), a member of the G protein-coupled receptor (GPCR) family. CXCR4 and its endogenous ligand C-X-C motif chemokine 12 (CXCL12) play a crucial role in various immune diseases and cancer progression (Borja et al. 2021; Bleul et al. 1997; Lee et al. 1999). A number of studies have verified the involvement of the activated CXCL12/CXCR4 axis in promoting growth, survival, and invasive capacity in solid cancers (Chatterjee et al. 2014; Bianchi and Mezzapelle 2020). In GBM, the signal transduction pathways and regulation of CXCR4 are essential for sustained invasion (Rubin et al. 2003), enhanced angiogenesis (Kioi et al. 2010), and the migration of glioma stem cells, leading to therapeutic resistance (Goffart et al. 2015). Additionally, the CXCR4 is upregulated under hypoxic conditions, which are associated with a poorer prognosis (Eckert et al. 2018).

**Fig. 1:**
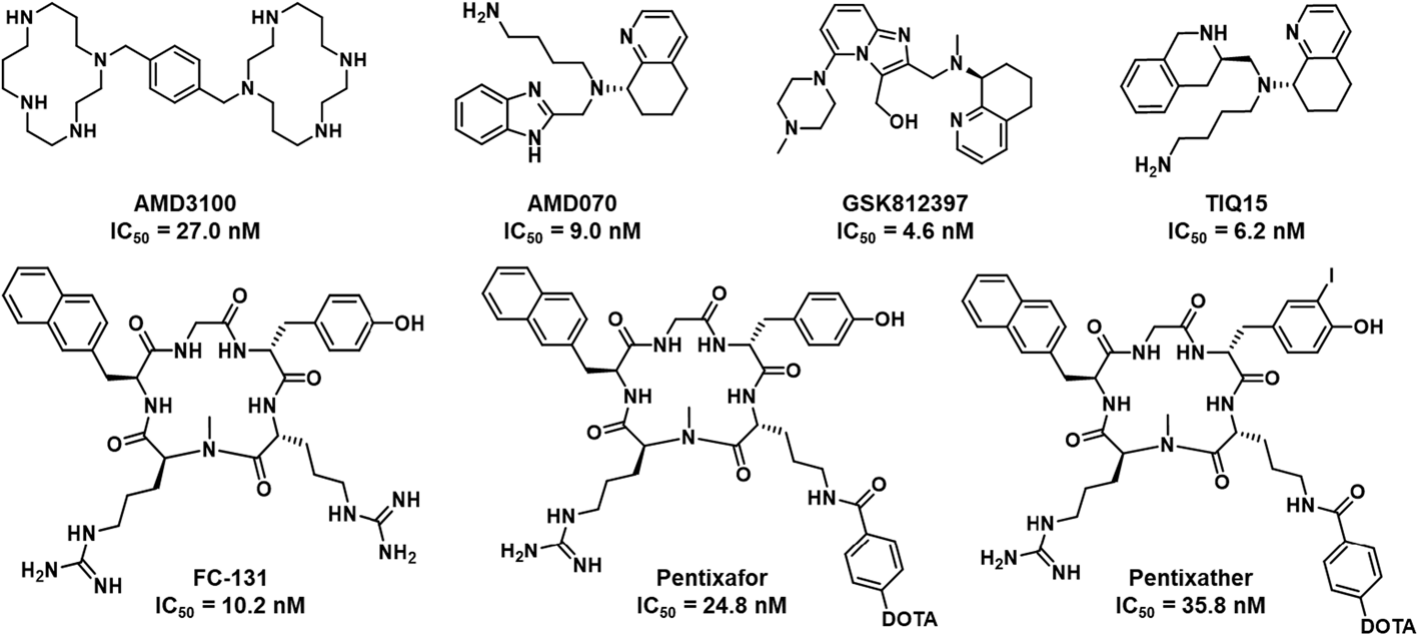
Chemical structures of CXCR4 antagonists and BFCs in clinical translation

The impact of the CXCL12/CXCR4 axis on promoting GBM growth has spurred the development of effective CXCR4 antagonists as potential anti-cancer drugs (Rubin et al. 2003; Kioi et al. 2010; Goffart et al. 2015; Eckert et al. 2018). Notably, a bicyclam AMD3100, (IC_50_ = 27.0 nM) became the first approved drug targeting CXCR4 (Fricker et al. 2006), while non-cyclam CXCR4 antagonists of various chemotypes were further developed including cyclic peptides (Ueda et al. 2007), modified cyclic peptides (Tamamura et al. 2005), tetrahydroisoquinoline derivatives (Truax et al. 2013; Skerlj et al. 2010; Mosi et al. 2012; Nyunt et al. 2008) and benzenesulfonamide (Oum et al. 2020). Among the highly potent CXCR4 antagonists, there are tetrahydroisoquinoline based compounds such as TIQ-15 (IC_50_ = 6.2 nM) (Truax et al. 2013), mavorixafor (AMD070, IC_50_ = 9.0 nM) (Skerlj et al. 2010) and GSK812397 (IC_50_ = 4.6 nM) (Jenkinson et al. 2010). While the structures of AMD3100 and a modified cyclic peptide, FC-131, (IC_50_ = 10.2 nM) have been exploited to be a tracer motif for diagnosis purpose (Thiele et al. 2014). For instance, [^68^Ga]Ga-AMD3100 has been clinical translated as a PET tracer for diagnosis of solid tumors (Renard et al. 2023). Currently, [^68^Ga]Ga-pentixafor (Dreher et al. 2024) is being studied in Phase 3 clinical trial for staging of marginal zone lymphoma (https://clinicaltrials.gov/study/NCT06125028?cond=NCT06125028&rank=1#publications). In addition to the diagnosis application, pentixather has been developed for targeted radiotherapy by labeling with ^177^Lu (Schottelius et al. 2017). [^177^Lu]Lu-pentixather is being studied in a Phase 2 clinical trial for CXCR4-positive acute leukemia (https://clinicaltrials.gov/study/NCT06356922?cond=NCT06356922&rank=1). The chemical structures of CXCR4 antagonists and the derived bifunctional chelators (BFCs) for radiolabeling under clinical translation are shown in Fig. 1.

This study aims to investigate the potential of the ^68^Ga-based CXCR4-PET tracer, [^68^Ga]Ga-TD-01, for brain cancer imaging, particularly GBM. Although the radiopharmaceuticals, [^68^Ga]Ga-pentixafor and [^177^Lu]Lu-pentixather, are currently in clinical trials, they are focused on lymphoma and acute leukemia and have not been studied in GBM. Our study is the first report of a novel Ga-68 based PET tracer targeting CXCR4 in an orthotopic mouse model of GBM (GL261). This follows our comparative studies of F-18 PET tracers for brain tumors (Lindemann et al. 2023). The orthotopic model is more clinically relevant because the tumors are implanted in their original anatomical location, providing a realistic tumor microenvironment. Previous studies of [^68^Ga]Ga-AMD3100 in GBM-bearing mice used the U87 xenograft model with subcutaneous injection of GBM cell lines in athymic mice (Renard et al. 2023), which is less accurate in replicating the native tumor environment in particular the immune response and behavior. Moreover, our study highlights the benefit of [^68^Ga]Ga-TD-01, which is easily synthesized and translatable to endoradiotherapy with radiometals, compared to F-18 based PET tracers for brain cancers, including [^18^F]rhPSMA-7.3, [^18^F]FET, and [^18^F]fluciclovine. Additionally, the orthotopic model is excellent for evaluating the delivery and efficacy of therapeutic agents, including their ability to cross the blood–brain barrier (BBB) and specifically target tumor cells within the brain.

## Materials and methods

### General

All chemical reagents and solvents were obtained from commercial sources (Combi-Blocks, USA, Oakwood Chemical, USA, Thermo Fisher Scientific, USA, Fisher Scientific, USA, and AmBeed, USA) and used without further purification unless otherwise noted. NMR spectra were recorded on a Bruker 400 MHz NMR spectrometer (Bruker, USA). Mass spectra for the small molecules were obtained using an Orbitrap Exploris 120 LC–MS (HRMS) instrument (Thermo Fisher Scientific, USA). Thin layer chromatography (TLC) was carried out on silica gel 60 (Merck, 230–400 mesh ASTM, Germany). HPLC was performed using Agilent 1260 Infinity II (Agilent Technologies, USA) with a XBridge C18, 4.6 × 150 mm, 5 µm HPLC column (Waters Corporation, USA).

### Chemical synthesis

The bifunctional chelator (BFC) was prepared by conjugating a CXCR4 antagonist, TIQ15 with the chelator (*p*-NCS-Bz-DOTA, CheMatech, France). The TIQ15 was synthesized in 5 steps as described previously (Truax et al. 2013). After that, TIQ15 (50 mg, 0.14 mmol) and *p*-NCS-Bz-DOTA (62 mg, 0.10 mmol) were dissolved in 1 mL of NH_4_OAc (pH 8) (Fisher Scientific, USA). The crude product was purified by reverse-phase SPE (Oasis C18 HLB plus short cartridge, Waters Corporation, USA). The cartridge underwent a wash sequence, initially with 5 mL of water followed by 8 mL of 10% ethanol. Subsequently, the compound was eluted using 3 mL of 100% ethanol, producing TD-01 in the form of white powder (32.15 mg, 61.18%). ^1^H-NMR: (400 MHz, MeOD) δ 8.54 (d, *J* = 5.0 Hz, 1H), 7.60 (d, *J* = 7.8 Hz, 1H), 7.48 (d, *J* = 8.5 Hz, 2H) 7.32 − 7.17 (m, 6H), 7.10 (m, 1H), 3.67 − 3.44 (m, 5H), 3.33 (s, 6H), 3.27 − 2.41 (m, 21H), 2.22 (m, 2H) 1.96 (m, 2H), 1.70 − 1.51 (m, 4H). ^13^C-NMR: (101 MHz, CDCl_3_) δ 181.15, 176.14, 174.53, 172.48, 162.18, 161.84, 131.52, 130.85, 129.01, 128.81, 128.06, 127.68, 127.37, 127.01, 126.40, 120.86, 121.07, 118.16, 115.25, 60.85, 56.09, 55.18, 54.39, 51.72, 45.35, 42.91, 42.49, 38.93, 38.68, 30.61, 30.31, 29.94, 27.04, 23.70, 22.33, 22.23, 20.18, 18.10. HRMS (ESI): m/z calcd for (C_50_H_69_N_10_O_9_S) 985.4975, found 985.4972 [M-H].

### Radiosynthesis of [^68^Ga]Ga-TD-01

The ^68^Ga^3+^ obtained from a ^68^Ge/^68^Ga generator (Eckert & Ziegler, Germany) was eluted with 0.1 N HCl, and the ^68^GaCl_3_ (~ 700 MBq in 1.5 mL of 0.1 N HCl) was transferred to the reaction vial containing the heating bifunctional chelator (TD-01) 50 µg in 1.5 mL of 0.1 M NH_4_OAc (pH 4.0) (Fisher Scientific, USA). The reaction was operated at 90 °C for 15 min. After 15 min, the reaction mixture was cooled to room temperature and transferred to the SPE cartridges (Oasis C18 HLB plus short cartridge, Waters Corporation, USA) for purification. The final product was wash with 10 mL of water and eluted from the HLB cartridge using 400 µL of 50% EtOH into the sterile product vial. After purification, the ethanol was removed and the product was formulated with 200 µL of normal saline for quality control and in vivo experiments. The radiochemical purity was determined by radio-TLC (silica gel 60 F_254_, aluminum sheet, mobile phase: MeOH:water = 6:4) and radio-HPLC. For HPLC, the following gradient between 0.1% TFA in ACN and 0.1% TFA in water was used at a flow rate of 1 mL/min: 0–2 min: 15% of 0.1% TFA in ACN, 2–5 min: 20% of 0.1% TFA in ACN, 5–7 min: 30% of 0.1% TFA in ACN, 7–10 min: 40% of 0.1% TFA in ACN, 10–17 min: 50% of 0.1% TFA in ACN, and 17–18 min: 15% of 0.1% TFA in ACN. The analysis was performed using a C18 HPLC column (XBridge C18, 4.6 × 150 mm, 5 µm, Waters, Corporation, USA). Radionuclidic impurities were determined using gamma spectroscopy with a Canberra system (Mirion Technologies, USA), which comprised a GX1520 high-resolution germanium detector (Mirion Technologies, USA), a DSA-1000 digital signal processor, and Genie 2000 software.

### In vitro stability studies

The stability of the produced PET tracer, [^68^Ga]Ga-TD-01 was evaluated in two different conditions, namely, 0.01 M phosphate buffered saline (PBS) pH 7.4 and human plasma (International Bio Service, Thailand). The radiolabeled compound (100–150 MBq) was incubated at 37 °C in two different conditions, and aliquots were taken at 1 h, 2 h, 3 and 4 h after the incubation. The radioactivity of intact radioligand was determined by a radio-TLC imaging scanner using a MeOH/water 3:2 solvent mixture. The stability of the produced PET tracer was reported by plotting %RCP versus time. The reported %RCP values were the average of three independent measurements.

### In vitro partition coefficient

A solution of [^68^Ga]Ga-TD-01 (100–150 MBq) was added in a mixture of 500 µL of PBS pH 7.4 and 500 µL of octanol. Vials were vortexed vigorously for 6 min and then were centrifuged at 14,600 rpm for 6 min to achieve quantitative phase separation. The radioactivity of the radiotracer in 100 µL samples of both the aqueous and the octanol phase were counted by 2470 WIZARD automatic gamma counter (Perkin Elmer, USA). The partition coefficient (P) was determined by P = (activity of radiolabeled compounds in octanol)/(activity of radiolabeled compounds in aqueous layer). Finally, log *P* was calculated, the reported log *P* value is the average of 3 independent determinations.

### Competition binding assay

The CXCL12^AF647^ binding assay with Jurkat cells (Thermo Fisher Scientific, USA) has been described previously (Schoofs et al. 2018), (Hout et al. 2017). Jurkat cells (ATCC, USA) were resuspended in Hank’s Balanced Salt Solution (HBSS) (Thermo Fisher Scientific, USA), 20 mM HEPES buffer (Thermo Fisher Scientific, USA), 0.2% bovine serum albumin (Sigma-Aldrich, USA), pH 7.4, at 3 × 10^5^ cells per sample and treated with different concentrations of TD-01 in HBSS at RT for 15 min. Afterwards, the cells were incubated with 2.9 nM CXCL12^AF647^ in HBSS at RT for 30 min in the dark. Cells were fixed in 1% paraformaldehyde in DPBS, and specific CXCL12^AF647^ binding, i.e., mean fluorescence intensity (MFI), was quantified by flow cytometry (BD FACSArray, Fisher Scientific, UK). Data were analyzed with FlowJo Software. The inhibitory concentration 50 (IC_50_) was calculated by using GraphPad Prism 10.0 (La Jolla, CA, USA) for TD-01 relative to the negative (i.e., autofluorescence of untreated and unlabeled cells) and positive (i.e., untreated cells exposed to CXCL12^AF647^ only) control.

### Animal studies

All animal experiments in this study received ethical approval from the Norwegian Food Safety Authority (Mattilsynet, #19743, 28409). Female C57BL/6JRj mice, aged 6–8 weeks, were obtained from Janvier (Le Genest-Saint-Isle, France). The mice were housed in groups of 4 and provided unrestricted access to food, water, and environmental enrichment. A period of 7 days was allocated for the animals to acclimatize before commencing the experimental procedures. The sample size for this study was determined a priori, with 4 animals per group considered sufficient to achieve a test power exceeding 90%.

### Syngeneic orthotopic animal model of GBM

The generation of the orthotopic mouse model was described elsewhere (Lindemann et al. 2023). Briefly, 8 female C57BL/6JRj mice, 6–8 weeks, 18–20 g were selected for subsequent surgery. Following isoflurane anesthesia (1.8% in O_2_), the mouse's head was shaved and fixed onto an automatized stereotactic frame (Neurostar, Germany). A skin incision was made using a scalpel, exposing the bregma and lambda landmarks and a hole drilled at − 2.3 mm lateral and − 0.5 mm anterior to the bregma. A 10 μL Hamilton syringe, filled with 3 μL of the cell suspension (GL261-luc2, 25000 cells/μL), was inserted 3 mm deep into the burr hole. Subsequently, 2 μL of the cell suspension was injected over 10 min. The syringe was then slowly removed, and the burr hole was sealed using bone wax, followed by suturing of the skin.

### Biodistribution of [^68^Ga]Ga-TD-01 in healthy mice using dynamic PET/MRI

Healthy C57BL/6JRj mice (n = 3) were used for biodistribution study via dynamic PET/MRI scans (MR solutions, Guildford, UK). The mice received an i.v. injection (lateral tail vein) of 7.2 ± 0.3 MBq [^68^Ga]Ga-TD-01. The anesthetized animals (1.8% isoflurane) were placed into a heated mouse imaging bed (Minerve, Équipement Vétérinaire MINERVE, France) with a respiratory sensor. A total of sixty minutes of dynamic PET (MR solutions, GB) was started simultaneously with radiotracer injection. The organs were manually delineated on the T1-weighted MRI and then superimposed with PET was performed in healthy animals for whole-body organ identification. Data analysis was performed by defining volumes of interest (VOI) of the major organs (PMOD, v.4.3, PMOD technologies, Switzerland). The time-activity data was expressed as the mean standardized uptake value (SUV) of the overall VOI.

### In vivo localization study of [^68^Ga]Ga-TD-01 in GBM-bearing mice using dynamic PET/MRI under baseline conditions

C57BL/6JRj mice (n = 4) bearing GL261-luc2 tumor received an i.v. injection of [^68^Ga]Ga-TD-01 6.3 ± 0.9 MBq and 60 min dynamic PET was started simultaneously. T1- and T2-weighted FSE high resolution MRI was performed simultaneously using a dedicated mouse head coil with the head fixed by ear bars and respiratory triggering to avoid movement artifacts. VOI data analysis was performed with PMOD according to the biodistribution study. Tumor tissue was defined on hyperintense MRI lesions of the T2-weighted images and co-registered with the dynamic PET. Tumor delineation was done manually based on hyperintense MRI signal in image and a similar-sized VOI (contralateral counterpart) was used for the healthy hemisphere (Fig. S1) as the control region to evaluate the tumor-to-background ratio (the ratio of the SUV_mean_ of the tumor VOI to the SUV _mean_ of the contralateral VOI: SUV_mean_Tumor/SUV_mean_Contralateral). The time-activity data was expressed as the mean standardized uptake value (SUV) of the overall VOI.

### In vivo localization study of [^68^Ga]Ga-TD-01 in GBM-bearing mice under blocking conditions

To determine the specificity of [^68^Ga]Ga-TD-01, a receptor-blocking study was performed to confirm that the developed PET radiotracer visualizes specifically CXCR4 by CXCR4 antagonist TIQ15. Therefore, TIQ15 (10 mg/kg in 100 µL of normal saline) was administered i.v. 10 min prior to radiotracer application (5.3 ± 1.3 MBq) and 60 min PET/MRI to investigate the reduction of the amount of the radiotracer due to competitive binding at the same receptor site. C57BL/6JRj mice bearing GL261-luc2 tumor (n = 3, tumor size ~ 0.05 cm^3^) were used for this experiment. The radiotracer uptake was measured by dynamic PET, and data was subsequently expressed as the mean standardized uptake value (SUV) of the overall regions of interest (ROI). The tumor-to-background ratio is a ratio of the SUV_mean_ of the tumor VOI to the SUV _mean_ of the contralateral VOI: SUV_mean_Tumor/SUV_mean_Contralateral brain tissue.

### Immunohistochemical analysis

For neuropathological analysis, brains from C57BL/6JRj mice bearing GL261-luc2 tumor were fixed in 4% paraformaldehyde (PFA) (Boster Bio, USA) and paraffine embedded (FFPE). 4 μm thick FFPE sections were incubated at 60 °C for 1 h, deparaffinized, and dehydrated. Antigen retrieval was performed by incubating the sections for 20 min at 97–98 °C with 10 mM sodium citrate buffer (pH 6.0). Subsequently, endogenous peroxidase activity was blocked with 3% H_2_O_2_ for 10 min. Sections were blocked for 20 min using 10% goat serum (Sigma-Aldrich, USA) in PBS, following incubation with primary antibody anti-CXCR4 [EPUMBR3] (Abcam, ab181020, 1:500) overnight at 4 °C. IHC staining was performed using the EnVision + System- HRP Labelled Polymer (Sigma-Aldrich, USA) and Liquid DAB + substrate chromogen visualization system (K346811-2) according to the manufacturer’s protocol. Finally, sections were counterstained with hematoxylin, rehydrated, and coverslips using the appropriate mounting medium (Histokitt, Chemi-Teknik AS, product no. 21412). Consecutive sections were stained using an H&E staining kit (Abcam, ab245880, UK). All reactions were performed at room temperature if not stated otherwise. Bright-field images were acquired using an upright Carl Zeiss Axio Imager.M2 equipped with an Axiocam 105 color camera operated with Zeiss ZEN 3.0 (blue ed.) software.

### Statistical analysis

The data analysis was performed using GraphPad Prism 10.0 (La Jolla, CA, USA). The numerical values are presented as mean ± standard deviation. The difference between groups was determined by a 2-tailed unpaired *t*-test. Statistical significance was considered when the *p*-value is < 0.05 (95% confidence level).

## Results

### Synthesis and chemical characterization

**Fig. 2:**
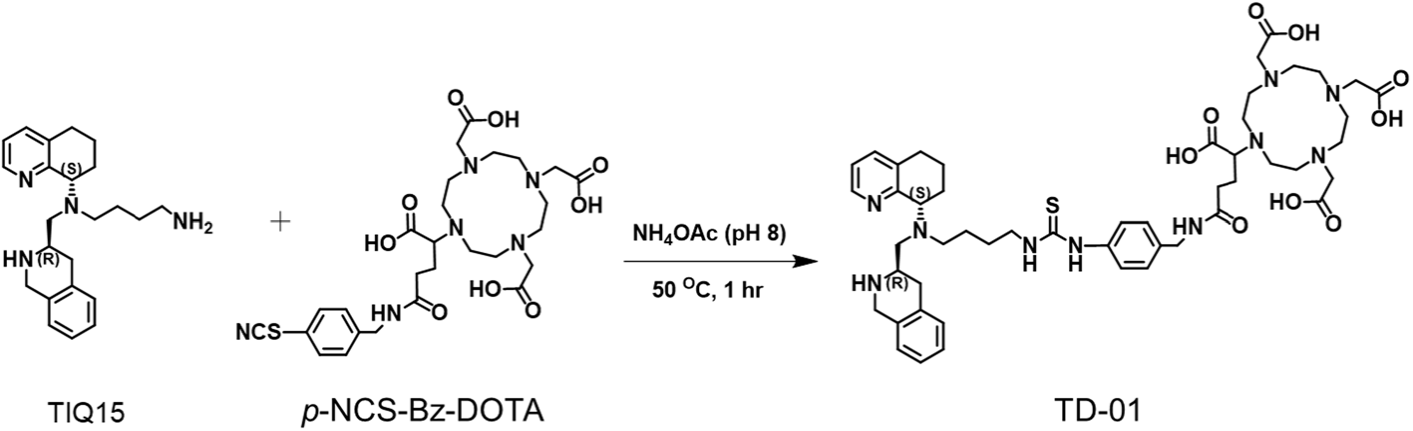
Synthesis of a bifunctional chelator, TD-01

The TIQ15 was synthesized in 5 steps using *N*-Boc (*R*)-2-(*tert*-butoxycarbonly)-1,2,3,4-tetrahydroisoquinoline-3-carboxylic acid as the starting material (Truax et al. 2013). After that, TIQ15 was conjugated with *p*-NCS-Bz-DOTA (Fig. 2). After purification by HLB SPE, 30 ± 3 mg amounts of TD-01 (68.92% yield) were obtained, with a purity of greater than 98%.

### Radiolabeling of TD-01 with ^68^Ga

**Fig. 3:**
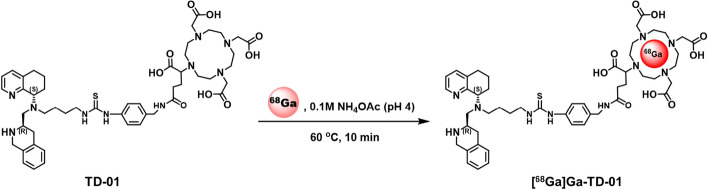
Radiosynthesis of [^68^Ga]Ga-TD-01

**Fig. 4:**
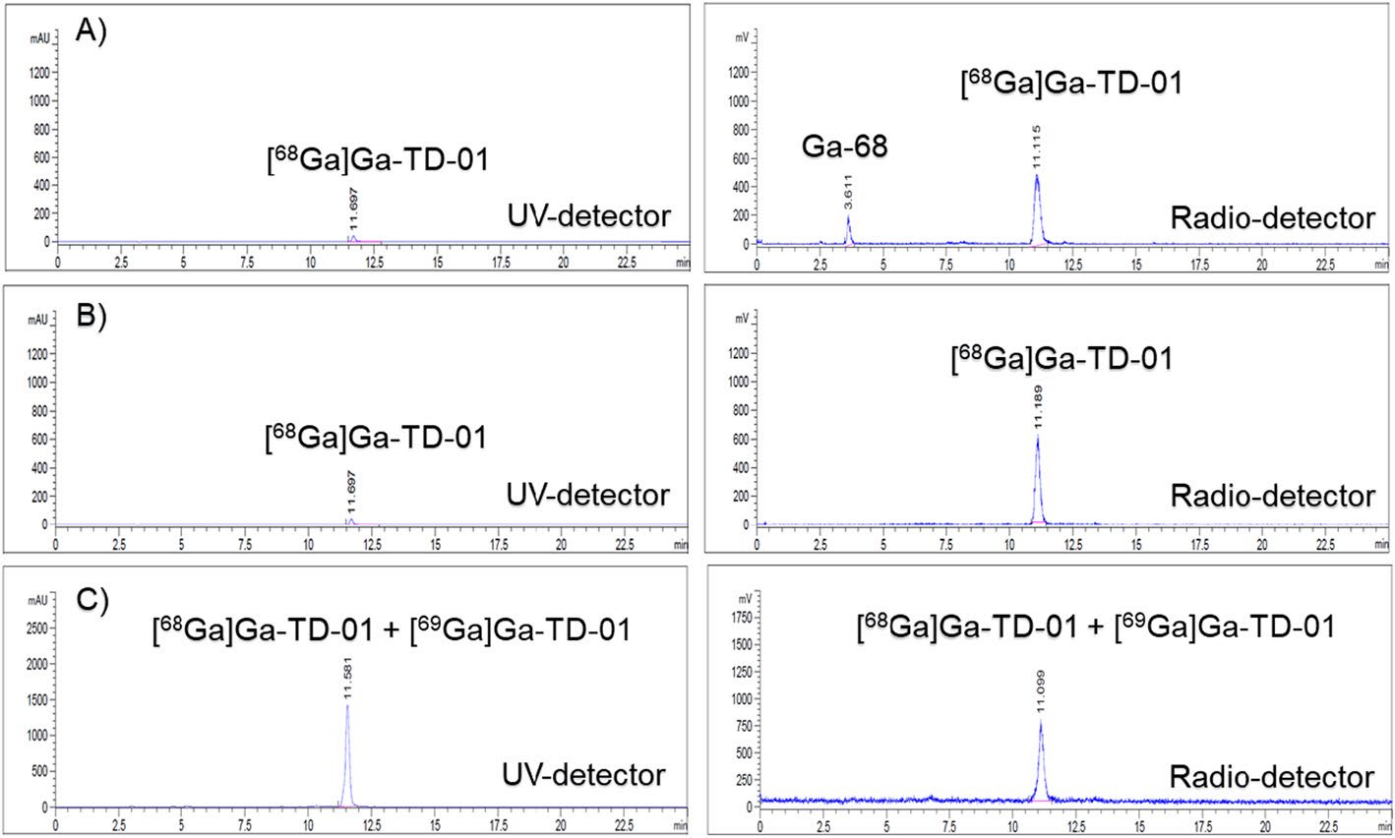
HPLC profile of [^68^Ga]Ga-TD-01. **A** crude product, **B** after purification and **C** spiked with non-radioactive—compound to confirm the product

The BFC bearing DOTA moiety for ^68^Ga complexation was successfully synthesized. Radiolabeling with ^68^Ga was performed under acidic conditions (pH 4) to produce a PET tracer ([^68^Ga]Ga-TD-01) (Fig. 3). After purification with a HLB SPE, the radiotracers were obtained with an overall radiochemical yield of 36.33 ± 1.50% (EOS). The purity of the final radiotracer was greater than 99% (analyzed by HPLC, Fig. 4), and the radioligand peak observed in HPLC chromatogram was further confirmed by co-injection of the corresponding non-radioactive compound. The radionuclidic purity was found to be > 99.9% (Fig. S7). The tested quality control parameters were reported in Table 1*.*

**Table 1:** Quality control of [^68^Ga]Ga-TD-01

Test parameters	[^68^Ga]Ga-TD-01 (n ≥ 3)
Radiochemical purity (RCP)	> 99%
Radiochemical yield (RCY)	36.33 ± 1.50% (EOS)
Molar activity (A_m_)	55.79 ± 1.96 GBq/µmol (EOS)
Appearance	Colorless solution
Radionuclidic purity	> 99.9%

### In vitro stability studies

**Fig. 5:**
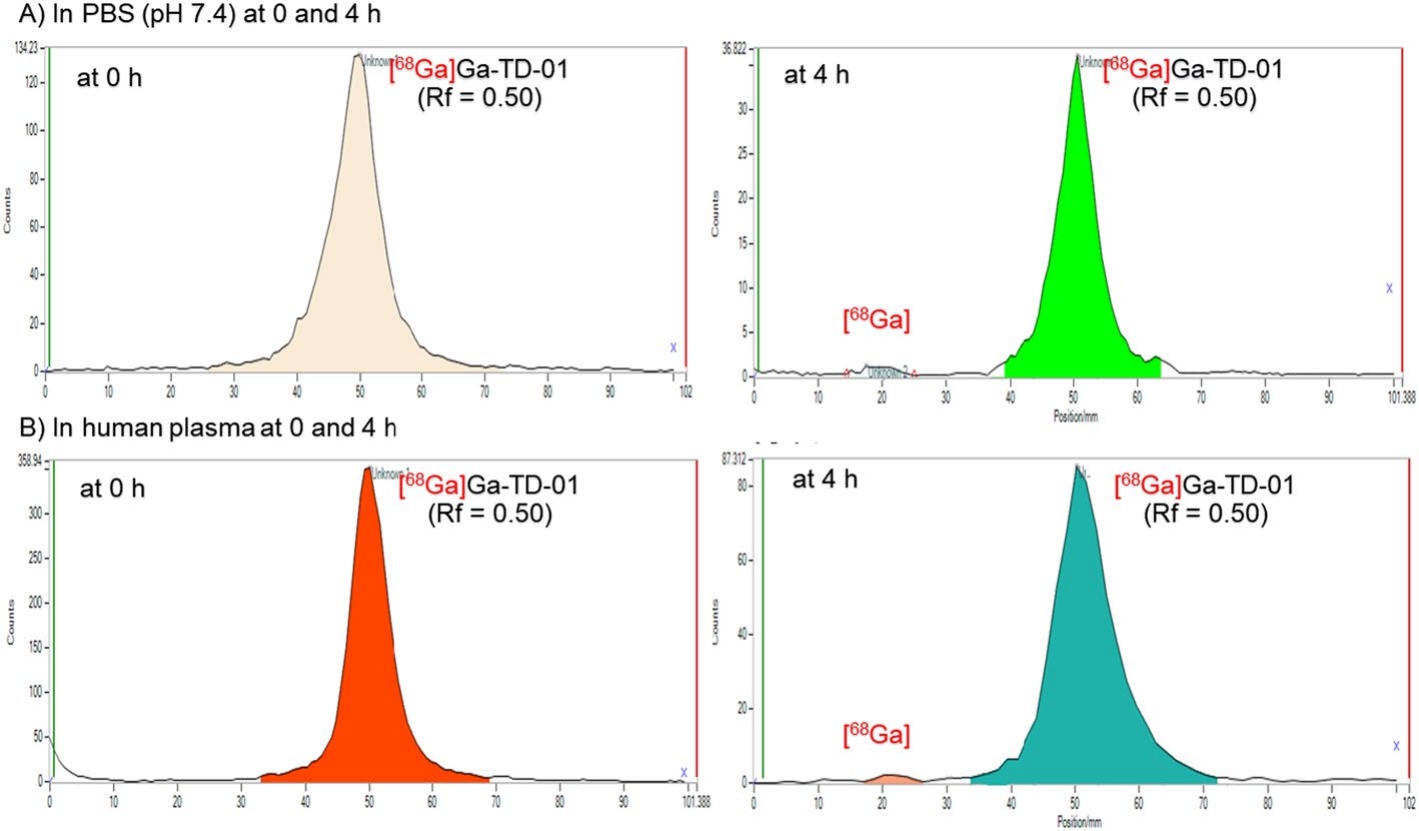
Radio-TLC chromatograms of [^68^Ga]Ga-TD-01. **A** Chromatograms of [^68^Ga]Ga-TD-01 in PBS (pH = 7.4) at 0 and 4 h. **B** Chromatograms of [^68^Ga]Ga-TD-01 in human plasma at 0 and 4 h

**Fig. 6:**
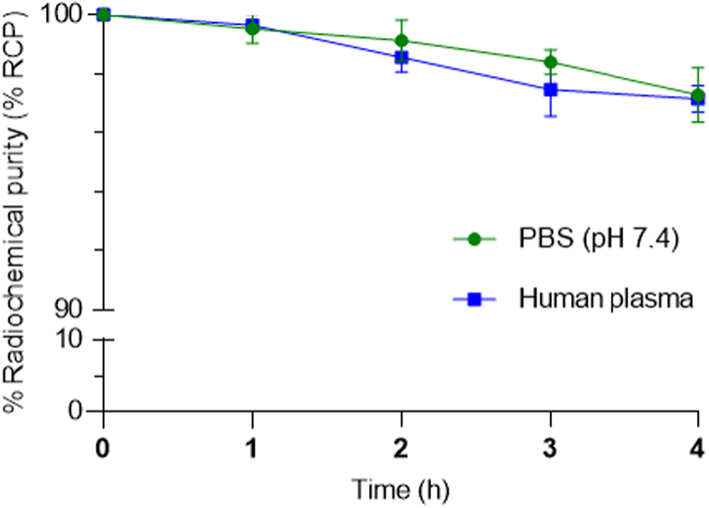
In vitro stability of [^68^Ga]Ga-TD-01 in PBS (pH = 7.4) and human plasma (n = 3)

The stability of [^68^Ga]Ga-TD-01 was determined at different time intervals by TLC, as described above. The radio-TLC chromatograms were depicted in Fig. 5. The radiolabeled complex remained stable at 1 h, 2 h, 3 h and 4 h for [^68^Ga]Ga-TD-01 post-incubation. Stability studies in two different conditions showed that > 95% of [^68^Ga]Ga-TD-01 remained intact up to 4 h post-labeling (Fig. 6). Results show high stability and suitability for in vivo experiments.

### In vitro evaluation of the binding characteristics of TD-01

**Fig. 7:**
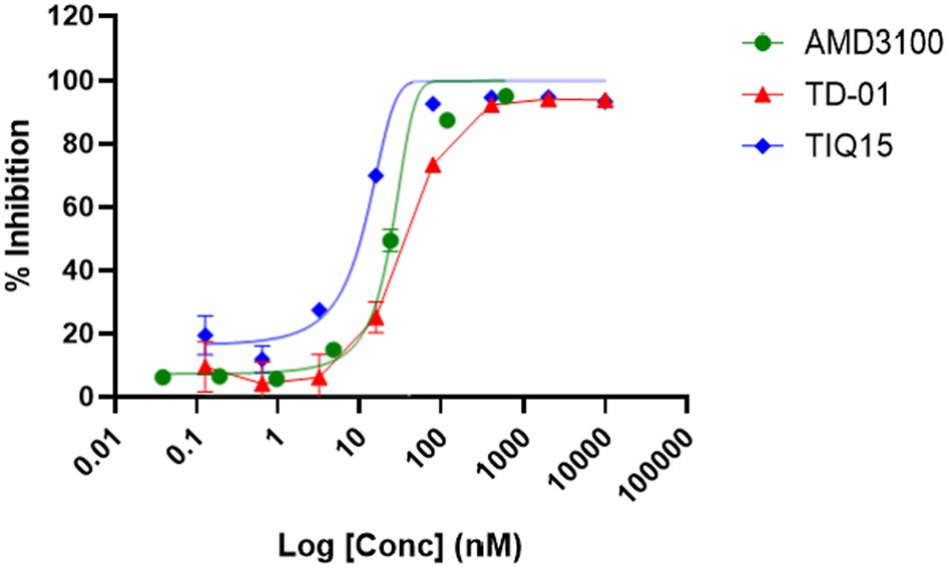
In vitro inhibition of CXCL12^AF647^ binding of TD-01 and related compounds on Jurkat CXCR4 + cells (n = 3)

The binding inhibition of the TD-01 for CXCR4 was evaluated with Jurkat cells, which the expression of CXCR4 receptors was confirmed by flow cytometric analysis (Fig. S6). The log concentration-inhibition curves for TD-01 and related compounds are shown in Fig. 7. TD-01 demonstrated an IC_50_ value of 36.50 nM against CXCR4-specific antagonist, TIQ15 (Table 2). As a positive control, we also tested the well-described CXCR4 antagonist, AMD3100, which demonstrated to have an IC_50_ of 24.70 nM. The IC_50_ value of TD-01 for CXCR4 was 36.5 nM, which is somewhat less potent than that of TIQ15 (IC_50_ = 7.5 nM).

**Table 2:** Inhibition of CXCL12^AF647^ binding of TD-01 and related compounds on Jurkat CXCR4 + cells (n = 3)

Compound	Inhibition of CXCL12^AF647^ binding (IC_50_, nM)	Binding inhibition from the literature (IC_50_, nM)
TIQ15	7.50 ± 0.4	6.20 (Truax et al. 2013)
TD-01	36.50 ± 1.4	N/A
AMD3100	24.70 ± 3.6	27.00 (Fricker et al. 2006)

### In vitro partition coefficient

The log *P* value of -1.06 ± 0.062 indicates that [^68^Ga]Ga-TD-01 is highly hydrophilic, similar to [^68^Ga]Ga-AMD3100 and [^68^Ga]Ga-pentixafor, which have log *P* values of − 1.95 (Renard et al. 2023) and -2.9 (Poschenrieder et al. 2016), respectively.

### PET/MRI of healthy and tumor-bearing animals

**Fig. 8:**
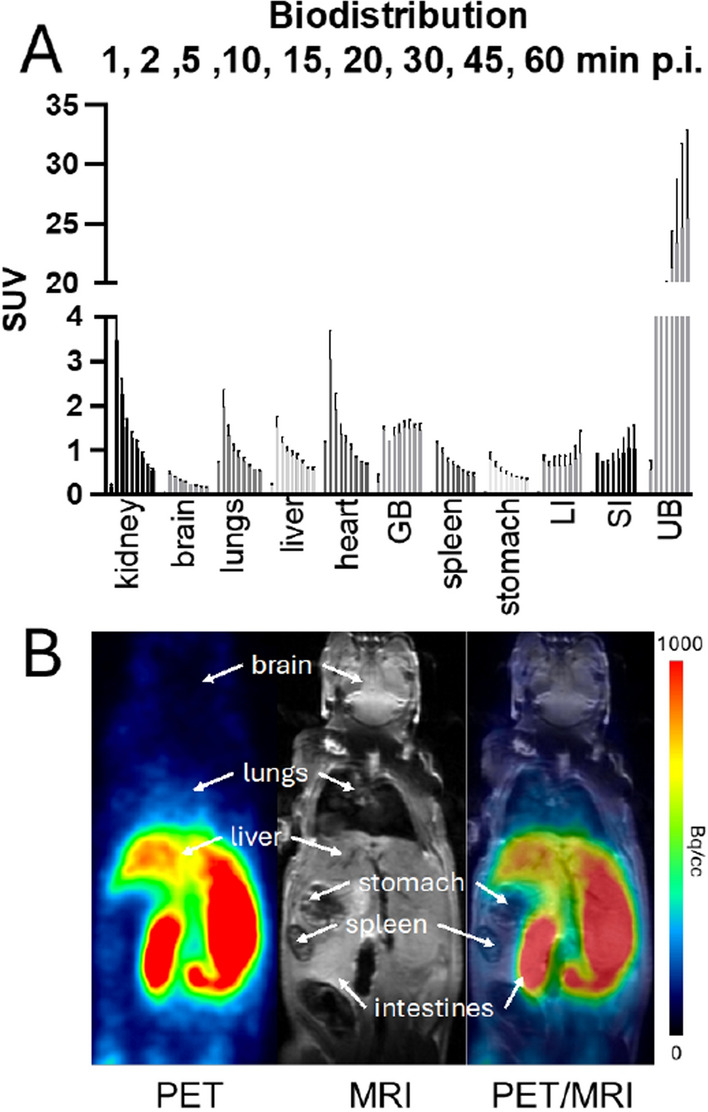
**A** Biodistribution of activity accumulation in major organs (SUV_mean_) derived from dynamic PET data in healthy C57BL/6JRj mice (n = 3) at 1, 2, 5, 10, 15, 20, 30, 45 and 60 min p.i. LI = large intestines, SI = small intestines, UB = urinary bladder, GB = gallbladder, **B** PET, T1-weighted MRI and PET/MR images at 60 min p.i

Following injection of [^68^Ga]Ga-TD-01, no adverse effects based on vital signs monitoring were observed during the investigation. At the end of the scan, the mice recovered fully within a short time. The PET images and dynamic data (Fig. 8) present high uptake in the kidney, liver, and urinary bladder, indicating a renal-hepatic radiotracer clearance. The moderate uptake was found in CXCR4 overexpressing organs like the lungs, spleen and intestines. Notably low uptake was observed in brain tissue of healthy mice with an approximate SUV of 0.5.

**Fig. 9:**
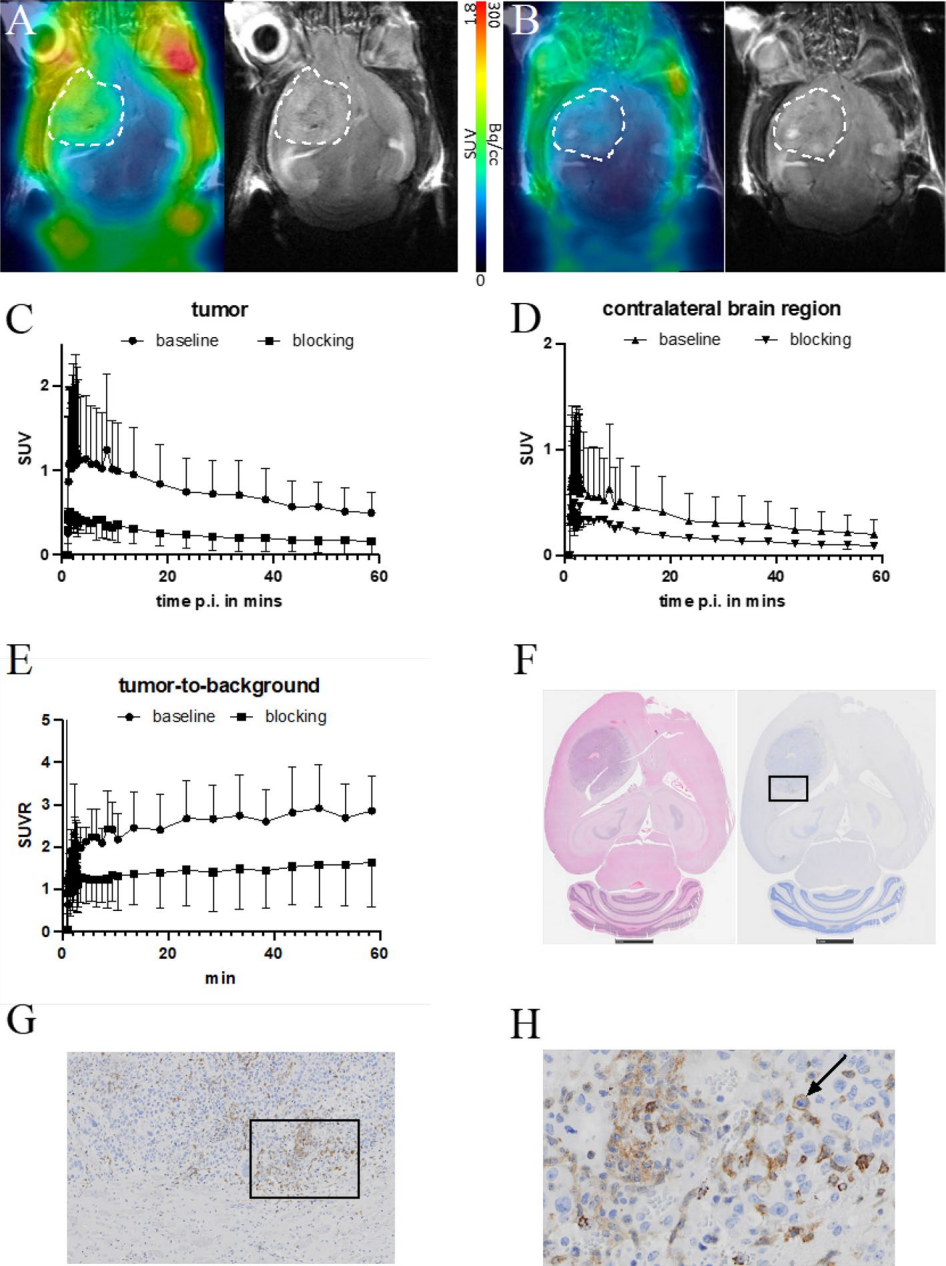
In vivo localization studies. 30 min PET/MR images of 2 separate animals under baseline **A** and blocking conditions with 10 mg/kg TIQ15, **B** following *i.v*. [^68^Ga]Ga-TD-01 showing a clear reduction in radiotracer uptake of the tumor region. The white dashed line indicates the tumor area. **C**, **D** Time-activity curves and **E** tumor-to-background ratio based on 1 h dynamic PET/MRI of tumor-bearing animals under baseline (n = 2) and blocking (n = 2) conditions for the tumor and the healthy brain region (SUV_mean_ ± standard deviation). **F** H&E staining (left) of a respective mouse brain with GL261-luc2 tumor and corresponding IHC staining against CXCR4 (right). The black box indicates the zoomed area in **G** or **H**, respectively. The arrow indicates a representative cell showing clear staining of the CXCR4 antibody

For GBM-bearing mice, low uptake in brain tissue (Figs. 9A, C, S2–S3) at early time points with SUV of 1.2 was observed. Subsequent blocking studies (Fig. 9B–E) in GL261-luc2-bearing animals using 10 mg/kg of the highly specific and selective compound TIQ15 10 min prior to PET were performed and showed a significant reduction in tumor uptake of [^68^Ga]Ga-TD-01. The statistical evaluation of dynamic SUV_mean_ values of the two groups (baseline vs. blocking) was calculated to be 0.93 (tumor baseline) and 0.35 (tumor blocking) (Fig. 9C). Hence, a reduction of signal of 62% (*p* < 0.0001) was observed (Fig. S4). The uptake of the radiotracer in the contralateral healthy brain was reduced, although to a lower extent (Fig. 9D). The SUV_mean_ values in healthy brain of the two groups (baseline vs. blocking) was calculated to be 0.53 (brain baseline) and 0.29 (brain blocking). Hence, a reduction of signal of 45% (*p* < 0.0001) was observed (Fig. S5).

For the tumor-to-background ratio, [^68^Ga]Ga-TD-01 uptake in glioblastoma (GBM)-bearing mice significantly decreased upon competition with TIQ15 in the blocking condition, with a baseline tumor-to-background ratios > 2.5 (20 min p.i.) (Fig. 9E), indicating high specificity. The IHC staining showed clear staining of the CXCR4 antibody indicating CXCR4 expression in the GBM model (Fig. 9F–H).

## Discussion

The development of effective diagnostic tools for early and accurate detection of brain tumors, particularly GBM, remains a pressing challenge in oncology. In this study, we present the development of a novel PET radiotracer, [^68^Ga]Ga-TD-01, targeting the CXCR4 for imaging GBM. This manuscript provides a comprehensive account of the synthesis, binding inhibition, radiolabeling, and preclinical evaluation of [^68^Ga]Ga-TD-01, offering valuable insights into its potential as a diagnostic tool for GBM. A novel bifunctional chelator (BFC), TD-01, was sought to be developed and designed through a rational drug design approach, not to change its authentic structure. However, introducing new labeling moieties such as a chelator (DOTA) in our novel tracer could potentially alter the biological properties of the radioligand. To assess these changes, we evaluated the binding inhibition (IC_50_) of the new compound (TD-01) compared to the original molecule (TIQ15) and a positive control (AMD3100). Our findings revealed that the IC_50_ value of TD-01 for CXCR4 was determined to be 36.5 nM, which is higher than that of TIQ15 (IC_50_ = 7.5 nM). Despite this fivefold reduction in the binding for CXCR4 compared to TIQ15, TD-01 still exhibited notable binding inhibition in the low nanomolar range, with an IC_50_ value (36.5 nM) comparable to those of AMD3100 (24.70 nM), pentixafor (24.8 nM) (Dreher et al. 2024), and pentixather (35.8 nM) (Schottelius et al. 2017).

Subsequent radiolabeling of TD-01 with ^68^Ga resulted in [^68^Ga]Ga-TD-01 with high radiochemical purity (> 99%) and favorable molar activity, indicative of its suitability for in vivo molecular imaging applications. The data from stability studies revealed that [^68^Ga]Ga-TD-01 remained intact (> 95%) up to 4 h post-labeling, both in PBS and human plasma, highlighting its robustness for in vivo applications.

Preclinical studies in healthy animals demonstrated favorable biodistribution and pharmacokinetics of [^68^Ga]Ga-TD-01, with prominent uptake in organs associated with CXCR4 expression, such as the spleen, intestines, and kidneys (Seemann and Lupp 2015; Zimmerman et al. 2011; Haege et al. 2012). The high uptake in the kidneys, liver, and urinary bladder corresponds to the hydrophilic log *P* value of [^68^Ga]Ga-TD-01 (− 1.06 ± 0.062), indicating a renal-hepatic radiotracer clearance. Regarding the uptake of [^68^Ga]Ga-TD-01 in lungs of healthy mice, it is plausibly due to the CXCR4 expression in this tissue (https://www.proteinatlas.org/ENSG00000121966-CXCR4/tissue). In normal healthy lung physiology, CXCR4 plays a key role in tissue repair and regeneration by mediating the homing and retention of progenitor cells. It is also involved in maintaining alveolar structure and function. The interaction of CXCR4 with its ligand, CXCL12, is essential for the immune response, aiding in the recruitment of immune cells to sites of inflammation or injury (Jaffar et al. 2020). The uptake of [^68^Ga]Ga-TD-01 in the lungs of healthy mice supported the crucial role of CXCR4 for tissue repair and regeneration. Concerning tissue-specific expression (https://www.proteinatlas.org/ENSG00000121966-CXCR4/tissue), CXCR4 is highly expressed in the bone marrow, where it plays a vital role in hematopoiesis. However, our study did not observe increased uptake of the radiotracer in bone. It is also worth noting that bone delineation is more challenging with the PET/MRI platform used in our study.

The dynamic PET/MRI scans in GBM-bearing mice confirmed specific uptake of [^68^Ga]Ga-TD-01 in tumor tissue. This was further validated by blocking studies with TIQ15, which demonstrated reduced tumor uptake upon competitive binding. IHC staining for CXCR4 in the brains of GBM-bearing mice confirm the presence of CXCR4 receptors on the cell membrane within tumor tissue. This validates the use of [^68^Ga]Ga-TD-01 as a PET radiotracer for visualizing CXCR4 expression in GBM. These findings align with previous reports of CXCR4 overexpression in various cancers, including GBM (Jiang et al. 2023). Importantly, the in vivo PET studies demonstrated that [^68^Ga]Ga-TD-01 can penetrate the BBB as shown in both healthy mice (biodistribution study) and mice with a glioblastoma model. In healthy mice, the SUV in brain tissue was approximately 0.5, which was comparable to that in the contralateral healthy brain tissue of GBM-bearing mice. However, in the GBM model with a permeable BBB, the tumor uptake had an increased SUV of 1.2, mainly due to high CXCR4 expression in the tumor, more than the permeable BBB caused by the tumor. This capability facilitates the imaging of CXCR4 expression in the brain. This information is highly relevant for interpreting in vivo data and determining the therapeutic and diagnostic efficacy of new compounds targeting CXCR4 receptors.

The role of CXCR4 in promoting tumor cell proliferation, invasion, and metastasis underscores its significance as a potential therapeutic target in GBM (Chatterjee et al. 2014). The development of [^68^Ga]Ga-TD-01 marks a promising advancement in GBM PET imaging, providing high specificity for detecting CXCR4 expression in tumor tissues. These studies collectively underscore the importance of CXCR4 as a promising biomarker for cancer imaging and highlight the potential of CXCR4-targeted PET tracers for non-invasive detection and characterization of malignancies. Indeed, the early and accurate detection of brain tumors, including GBM, is crucial. Regarding CXCR4 imaging, while its expression in GBM is not ubiquitous and is limited to a subset of cases (Jacobs et al. 2022), this specificity offers unique opportunities for targeted applications. CXCR4 imaging for glioblastoma can be particularly valuable in several ways: (*i*) Identifying patients whose tumors express CXCR4 could aid in stratifying treatment approaches, potentially guiding the use of CXCR4-targeted therapies. (*ii*) Monitoring response to therapy as CXCR4 expression may change with disease progression or in response to treatment. Imaging could provide real-time assessment of treatment efficacy and tumor response. (*iii*) Studying CXCR4 expression patterns can deepen our understanding of GBM heterogeneity and its implications for tumor behavior and treatment outcomes. Furthermore, we acknowledge the need for further research to define the precise roles and benefits of CXCR4 imaging in GBM management, considering its nuanced expression patterns.

The study bears some limitations, and the results should be interpreted in this regard. (*i*) The preclinical evaluation of [^68^Ga]Ga-TD-01 involved a relatively small sample size, which may limit the generalizability of the findings. Future studies with larger cohorts could provide more robust evidence of [^68^Ga]Ga-TD-01 efficacy and safety. Furthermore, due to the large tumor mass in the brain, the placement of the VOI might include signal from the first/second ventricle. However, a reference region of the same size was placed in the same contralateral position and will include the same signal and thus compensate for any bias. (*ii*) Stability studies showed favorable in vitro stability of [^68^Ga]Ga-TD-01 over 4 h. However, studies regarding in vivo stability have to confirm the suitability of the newly developed radiotracer. (*iii*) While the preclinical evaluation of [^68^Ga]Ga-TD-01 demonstrates its potential as a diagnostic tool for GBM, its clinical translation remains a significant hurdle. Further studies, including toxicity assessments and dosimetry calculations, are necessary to evaluate the safety and efficacy of [^68^Ga]Ga-TD-01 in clinical settings. (*iv*) Since our study observed low uptake in brain tissue of healthy mice, further investigation of the BBB status and the mechanism of BBB penetration are necessary. These can include dynamic contrast-enhanced MRI, PET imaging with BBB-specific tracers or transporters, and analysis of cerebrospinal fluid samples (Lindberg et al. 2023), (Vraka et al. 2018). (*v*) While [^68^Ga]Ga-TD-01 exhibited high affinity for CXCR4 and specificity in GBM-bearing mice, potential off-target effects and non-specific binding in other tissues cannot be ruled out. Additional investigations, such as competitive binding assays with other receptors and in vivo receptor blocking studies using heterologous competitive binding approaches, are needed to comprehensively elucidate the [^68^Ga]Ga-TD-01 specificity profile. The blocking studies using different CXCR4 antagonists could provide deeper insights into binding modes as the complexity of CXCR4 binding site, comprising both a large pocket and a smaller one. It implies that different CXCR4 antagonists, particularly those with lower molecular weight that may target different pockets, could present a more exhaustive validation and deeper insights into binding modes and affinities.

## Conclusion

In conclusion, the novel CXCR4 targeting PET radiotracer, [^68^Ga]Ga-TD-01, demonstrated high affinity for CXCR4, along with excellent in vitro stability and favorable pharmacokinetics. The substantial tumor uptake in GBM-bearing mice further indicates its specificity and potential application in brain cancer diagnostics. These encouraging results suggest that [^68^Ga]Ga-TD-01 could be effectively translated into clinical practice for in vivo characterization of CXCR4 expression in GBM and other CXCR4-implicated cancers, with significant potential for improving cancer diagnosis.

Future studies will further validate [^68^Ga]Ga-TD-01, including human tissue and full pharmacokinetic modeling, to assess its diagnostic efficacy and further develop it as a tool for brain cancer imaging with PET. Additionally, exploring its therapeutic potential by labeling it with therapeutic radionuclides, such as targeted drug delivery or combination therapy with existing treatments, could enhance its clinical utility in GBM management.

### Supplementary Information

Supplementary Information.

## Data Availability

The datasets generated during and/or analyzed during the current study are available from the corresponding author on reasonable request.

## Abbreviations

[^18^F]: Fluorine-18
[^68^Ga]Ga: ^68^Ga-gallium
[^68^Ge]: Germanium-68
[^177^Lu]Lu: ^177^Lu-lutetium
A_m_: Molar activity
BBB: Blood brain barrier
BFC: Bifunctional chelator
Bq: Becquerel
CXCL12: C-X-C motif chemokine ligand 12
CXCR4: C-X-C chemokine receptor 4
DOTA: 2,2′,2″,2‴-(1,4,7,10-Tetraazacyclododecane-1,4,7,10-tetrayl)tetraacetic acid
EOS: End of synthesis
FAP: Fibroblast activation protein
FDG: Fluorodeoxyglucose
FET: Fluoro-ethyl-tyrosine
FFPE: Paraffine embedded
GBM: Glioblastoma
GPCR: G protein-coupled receptor
H&E: Haematoxylin & eosin
HBSS: Hank’s balanced salt solution
HLB: Hydrophilic-lipophilic balanced
HPLC: High performance liquid chromatography
HRMS: High-resolution mass spectrometry
IHC: Immunohistochemistry
MFI: Mean fluorescence intensity
MRI: Magnetic resonance imaging
NMR: Nuclear magnetic resonance spectrometer
PBS: Phosphate buffered saline
PET: Positron emission tomography
*p*-NCS-Bz-DOTA: 2,2′,2″-(10-(1-Carboxy-4-((4-isothiocyanatobenzyl)amino)-4-oxobutyl)-1,4,7,10-tetraazacyclododecane-1,4,7-triyl)triacetic acid
PRT/MRI: Positron emission tomography/magnetic resonance imaging
PFA: Paraformaldehyde
PSMA: Prostate-specific membrane antigen
RCY: Radiochemical yield
RCP: Radiochemical purity
Rf: Relative retardation factor
ROI: Regions of interest
SPE: Solid phase extraction
SUV: Standardized uptake value
TLC: Thin layer chromatography
t_r_: Retention time
VOI: Volumes of interest
