# Supplementary material for: Radiosynthesis and preclinical evaluation of a 68Ga-labeled tetrahydroisoquinoline-based ligand for PET imaging of C-X-C chemokine receptor type 4 in an animal model of glioblastoma
Source: EJNMMI Radiopharm Chem. 2024 Aug 20;9:61. doi: 10.1186/s41181-024-00290-y (PMC11335985; doi:10.1186/s41181-024-00290-y)
Supplement: Supplementary file 1 — Supplementary Information. [file 41181_2024_290_MOESM1_ESM.docx]

Radiosynthesis and Preclinical Evaluation of a ^68^Ga-labeled Tetrahydroisoquinoline-based Ligand for PET Imaging of C-X-C Chemokine Receptor Type 4 in an Animal Model of Glioblastoma

Piyapan Suwattananuruk ^1,2^, Sukanya Yaset ^3^, Chanisa Chotipanich ^3^, Angel Moldes-Anaya ^4^, Rune Sundset ^4,5^, Rodrigo Berzaghi^5^, Stine Figenschau^5^, Sandra Claes^6^, Dominique Schols^6^, Pornchai Rojsitthisak ^1,2^, Mathias Kranz ^4†*^, Opa Vajragupta ^1,2†*^

| Contents | |  |
| --- | --- | --- |
| List of Fig | | Page |
| **Fig S1** | VOI placement, image showing the manual ROI placement of blue: the tumor and red: the contralateral region matching the tumor size. | 2 |
| **Fig. S2** | SUV mean and standard deviation during the first 15 minutes presenting moderate brain uptake indicating BBB penetration. | 2 |
| **Fig. S3** | Single values 0 to 4 minutes following radiotracer injection showing peak brain uptake of SUV 1.22 indicating BBB penetration. | 3 |
| **Fig. S4** | The two tailed unpaired t test of dynamic SUVmean values, the mean of the two groups (baseline vs blocking) was calculated to be 0.93 (tumor baseline) and 0.35 (tumor blocking) which is significant different from each other (*p*<0.0001). | 4 |
| **Fig. S5** | The two tailed unpaired t test of dynamic SUVmean values in healthy brain, the mean of the two groups (baseline vs blocking) was calculated to be 0.53 (brain baseline) and 0.29 (brain blocking). Hence a reduction of signal of 45 % was observed with a *p* value of <0.0001. | 5 |
| **Fig. S6** | Flow cytometry analysis of Jurkat cells stained with CXCR4 antibody (Clone 12G5). | 6 |
| **Fig. S7** | Gamma spectrum of Ga-68 sample. The gamma spectrum displays the energy distribution of counts obtained from a Ga-68 sample. The measurement was conducted over a live time of 3496.241 seconds and a real time of 3600.000 seconds. The prominent peak at approximately 511 keV corresponds to Ga-68. Additional smaller peaks are also identified at various energies. The spectrum indicates a high radionuclidic purity for Ga-68, as evidenced by the dominant peak at the expected energy for Ga-68 emissions. Data were collected using a high-purity germanium (HPGe) detector | 7 |


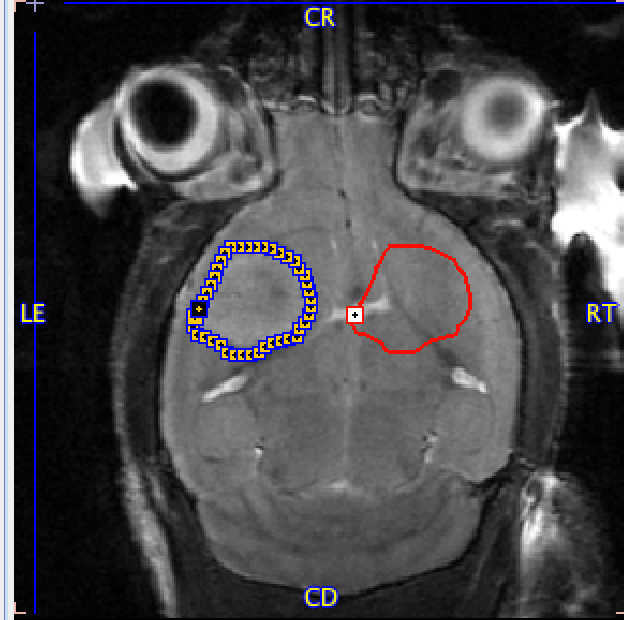


**Fig. S1** VOI placement, image showing the manual ROI placement of blue: the tumor and red: the contralateral region matching the tumor size.

**Fig. S2** SUV mean and standard deviation during the first 15 minutes presenting moderate brain uptake indicating BBB penetration.

**Fig. S3** Single values 0 to 4 minutes following radiotracer injection showing peak brain uptake of SUV 1.22 indicating BBB penetration.


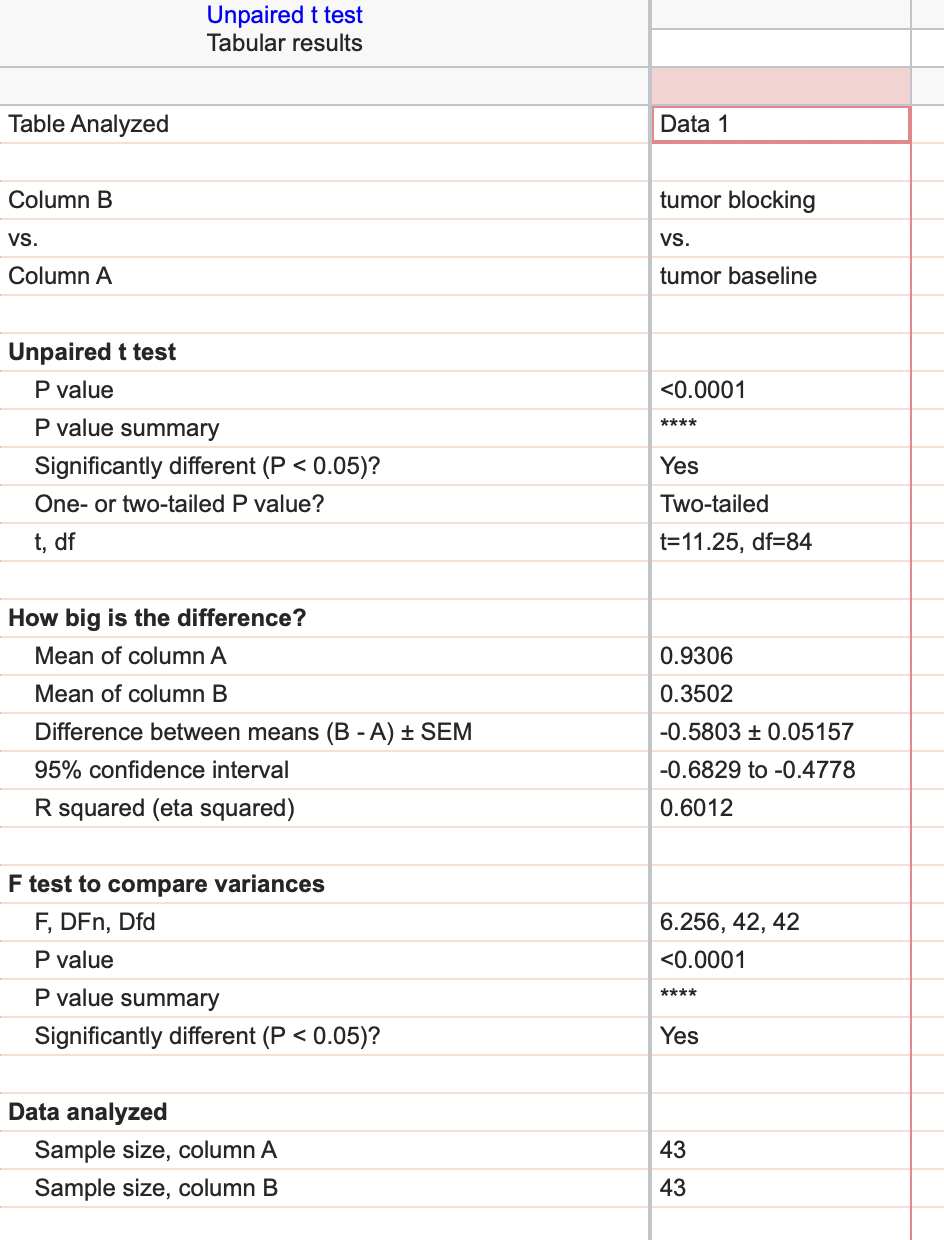


**Fig. S4** The two tailed unpaired t test of dynamic SUVmean values, the mean of the two groups (baseline vs blocking) was calculated to be 0.93 (tumor baseline) and 0.35 (tumor blocking) which is significant different from each other (*p*<0.0001).


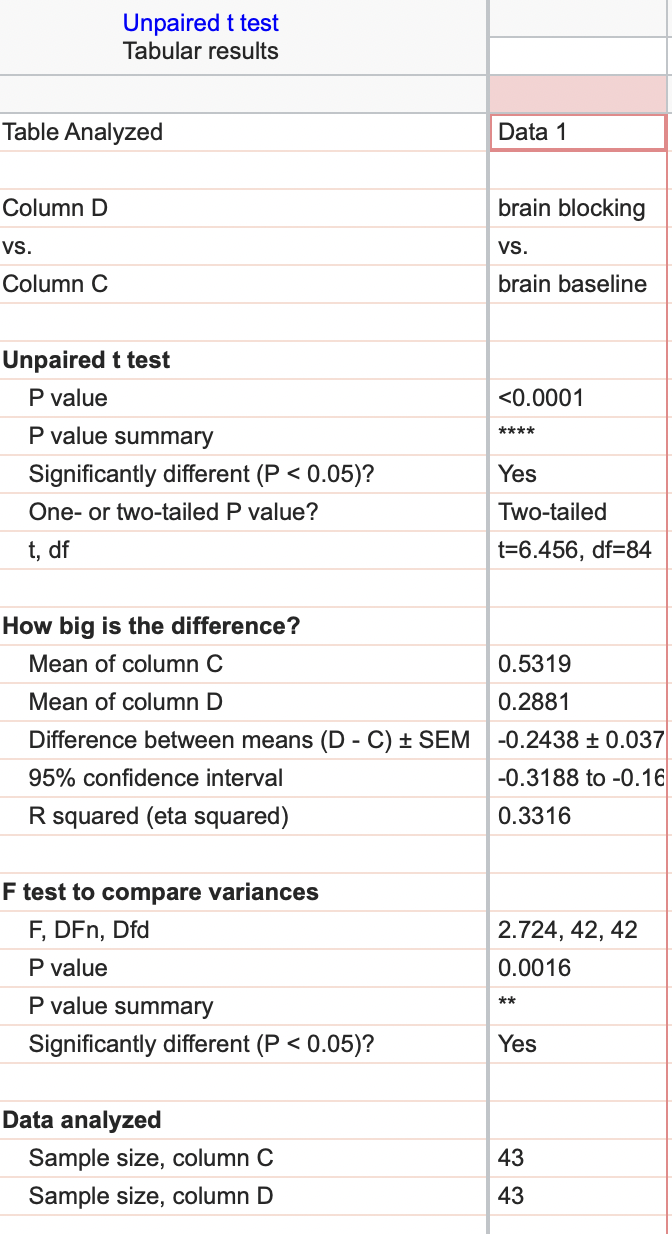


**Fig. S5** The two tailed unpaired t test of dynamic SUVmean values in healthy brain, the mean of the two groups (baseline vs blocking) was calculated to be 0.53 (brain baseline) and 0.29 (brain blocking). Hence a reduction of signal of 45 % was observed with a *p* value of <0.0001.


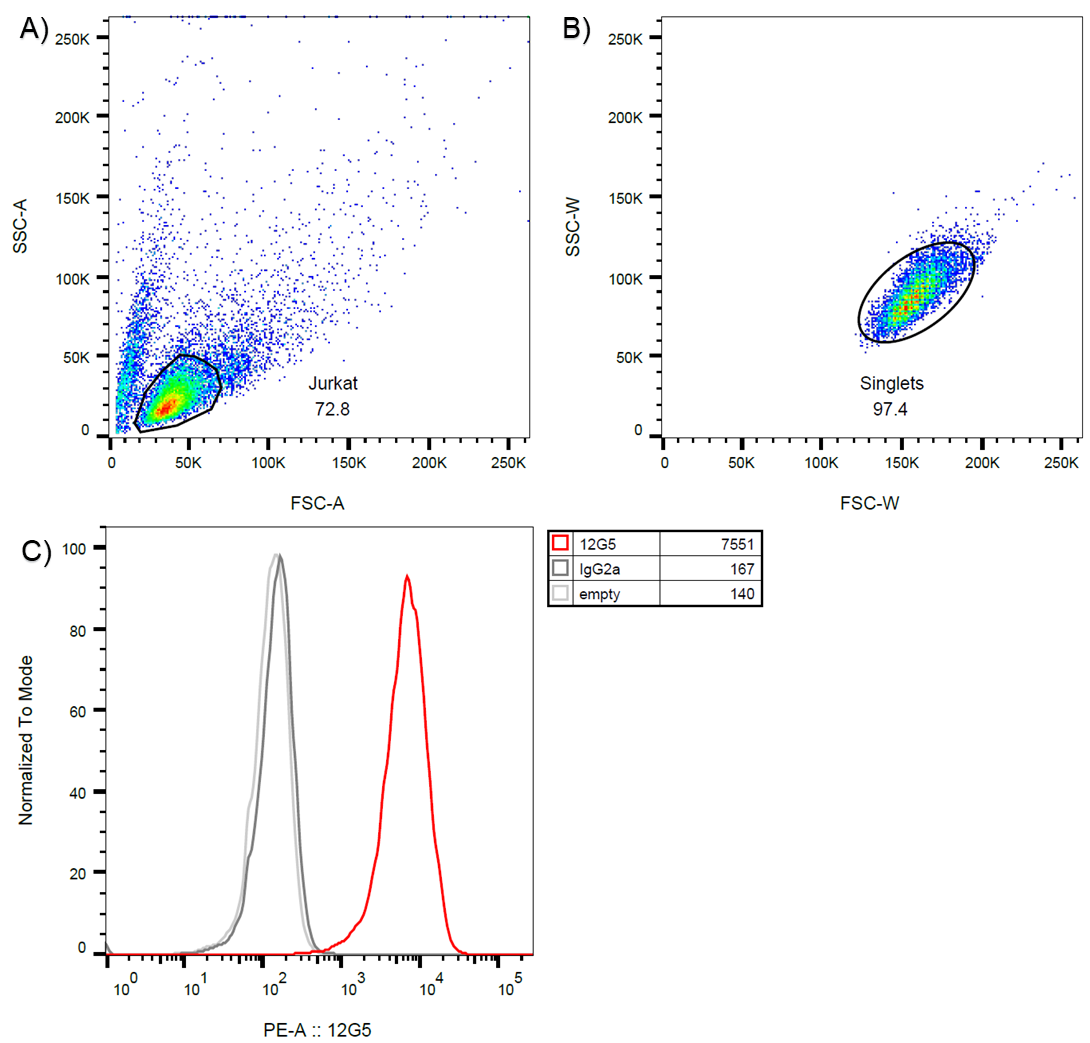


**Fig. S6** Flow cytometry analysis of Jurkat cells stained with CXCR4 antibody (Clone 12G5). The flow cytometry results show the analysis of Jurkat cells stained with CXCR4 antibody (clone 12G5). (A) The plot of SSC-A vs. FSC-A displays the side scatter area (SSC-A) versus the forward scatter area (FSC-A), identifying the Jurkat cell population, which represents 72.8% of the total events. (B) The plot of SSC-W vs. FSC-W isolates singlets, with 97.4% of the events falling within the singlet gate. (C) The plot shows the fluorescence intensity of PE-A for three samples: 12G5-stained cells (red), IgG2a isotype control (black), and empty control (gray). The mean fluorescence intensity (MFI) values are 7551 for 12G5, 167 for IgG2a, and 140 for the empty control, indicating specific binding of the 12G5 antibody to the CXCR4 receptor on Jurkat cells.


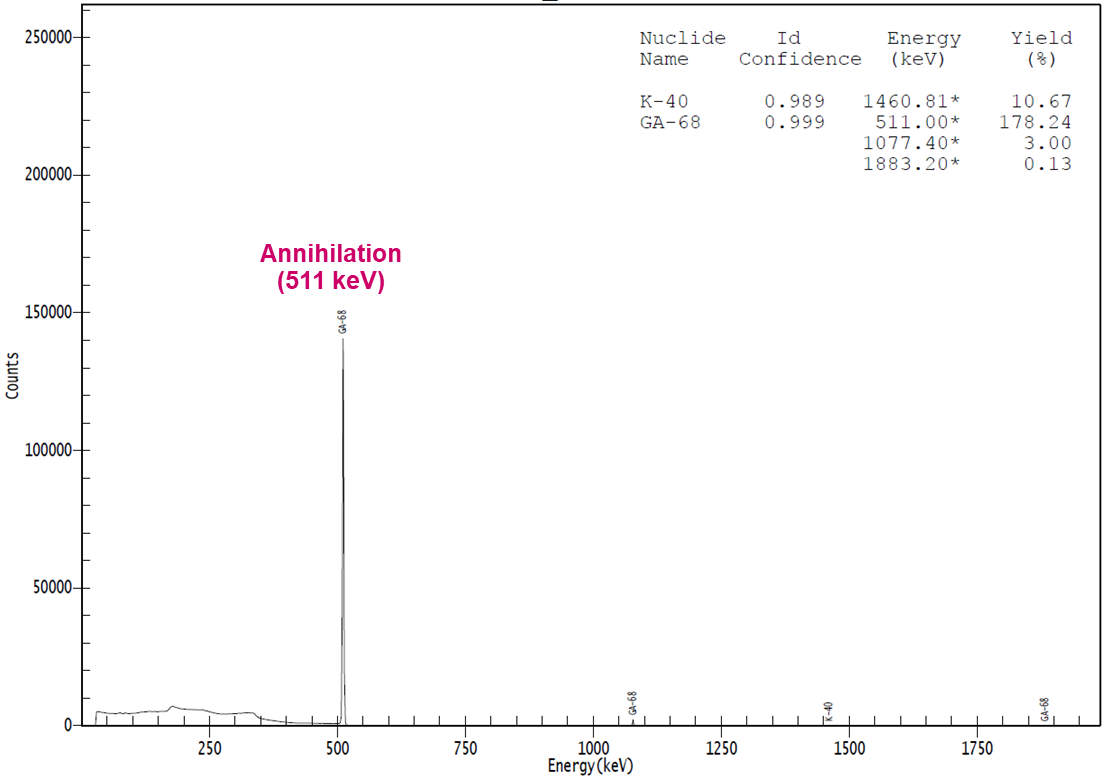


**Fig. S7** Gamma spectrum of Ga-68 sample. The gamma spectrum displays the energy distribution of counts obtained from a Ga-68 sample. The measurement was conducted over a live time of 3496.241 seconds and a real time of 3600.000 seconds. The prominent peak at approximately 511 keV corresponds to Ga-68. Additional smaller peaks are also identified at various energies. The spectrum indicates a high radionuclidic purity for Ga-68, as evidenced by the dominant peak at the expected energy for Ga-68 emissions. Data were collected using a high-purity germanium (HPGe) detector
